# Combating the Growing Threat of *Acinetobacter baumannii* Resistance

**DOI:** 10.3390/antibiotics14070694

**Published:** 2025-07-09

**Authors:** Eduarda Kffuri Goncalves, William Frank Penwell, Steven Eugene Fiester

**Affiliations:** 1 Department of Biological Sciences, Florida Gulf Coast University, Fort Myers, FL 33965, USA; ekkffurigoncalve6643@eagle.fgcu.edu; 2 Department of Biology and Marine Science, Jacksonville University, Jacksonville, FL 32211, USA; wpenwel@ju.edu; 3 Family Medicine Residency Program at Lee Health, Florida State University College of Medicine, Fort Myers, FL 33901, USA; 4 Internal Medicine Residency Program at Lee Health, Florida State University College of Medicine, Cape Coral, FL 33991, USA; 5 Department of Chemistry, Furman University, Greenville, SC 29617, USA; 6 School of Health Research, Clemson University, Clemson, SC 29634, USA; 7 Department of Pathology, Prisma Health Upstate, Greenville, SC 29601, USA

## 1. Introduction

Antimicrobial resistance remains one of the most urgent and complex challenges facing global public health in the 21st century. Among antibiotic resistant bacteria, *Acinetobacter baumannii* is considered a particularly alarming pathogen due to its propensity for drug resistance, making this bacterium a significant threat to patient safety and hospital infection control efforts [1]. Due to its resistance capability, *A. baumannii* is classified as one the ESKAPE pathogens, a group of six highly drug-resistant bacterial pathogens notorious for their ability to evade the effects of antimicrobial agents as well as the host immune response [2].

*A. baumannii* itself is a Gram-negative, aerobic bacterium commonly isolated in the clinical environment and generally associated with ventilator-associated pneumonia, bloodstream infections, urinary tract infections, meningitis, and wound infections in critically ill or immunocompromised patients [3]. The success of *A. baumannii* as a pathogen is, however, not solely due to its resistance phenotypes but also due to its ability to evade immune responses, tolerate desiccation, resist disinfectants, and form biofilms on surfaces and medical devices [4]. Taken together, these phenotypes compounded by this pathogen’s genetic plasticity make *A. baumannii* uniquely suited to thrive in healthcare environments. The global rise in multidrug-resistant (MDR), extensively drug-resistant (XDR), and pandrug-resistant (PDR) *A. baumannii* strains highlights an urgent need for innovative treatment options, deeper insights into resistance mechanisms, and strengthened international surveillance initiatives.

In recognition of the growing threat of drug-resistant *A. baumannii*, the World Health Organization has designated carbapenem-resistant *A. baumannii* (CRAB) as a critical priority pathogen for research and drug development due to its increasing prevalence and severe impact in healthcare settings worldwide [5]. This classification underscores the necessity for a coordinated response to mitigate this pathogen’s spread and improve treatment outcomes. *A. baumannii* has demonstrated an exceptional capacity to resist antimicrobial treatments. Its arsenal of resistance determinants includes the production of *β*-lactamases (particularly oxacillinases), the use of multidrug efflux pumps, alterations in outer membrane permeability, and the capacity to form biofilms. These resistance mechanisms do not act in isolation; rather, they interact synergistically, conferring high levels of resistance to diverse antibiotics, including last-resort agents like colistin [6].

The eight contributions included in this Special Issue collectively highlight the multifaceted approaches required to address *A. baumannii*, emphasizing the value of genomic surveillance, stringent infection control practices, strategic antimicrobial stewardship, and standardized testing for disinfectant resistance. Collectively, these contributions explore key dimensions of the *A. baumannii* challenge, including its antibiotic resistance mechanisms, the role of disinfectant protocols in outbreak prevention, the emergence of tolerance to biocidal agents, the influence of global clones on resistance phenotypes, and the assessment of current and experimental therapies, both as monotherapies and in combination regimens.

## 2. Overview of Published Papers

Genomic surveillance has proven essential for understanding the distribution and evolution of antimicrobial resistance. The article by Hamed et al. (contribution 1) conducted a comprehensive genomic study in Egypt, which revealed a distribution of global clones (GCs) among clinical isolates of *A. baumannii*, with global clone 2 (GC2) emerging as the dominant lineage in the resistome landscape. Isolates within this lineage demonstrated consistent resistance to multiple antibiotics, including amikacin, colistin, and trimethoprim. Further genomic analysis identified 51 resistance determinants across 10 antimicrobial classes. Genes such as *bla*_OXA-23_, *bla*_NDM-1_, and *armA* were widespread, while mutations in *carO* correlated strongly with carbapenem resistance. Among the resistance-associated genes, *bla*_OXA-23_ was the most prevalent and a key driver of resistance in clinical isolates. These findings underscore the importance of whole-genome sequencing in mapping resistance patterns and tailoring region-specific treatment and prevention strategies.

Beyond intrinsic genetic resistance, *A. baumannii* exhibits adaptive response mechanisms to environmental stressors, a feature that further complicates clinical management. One such mechanism, known as adaptive efflux-mediated resistance, enables the bacterium to upregulate efflux pump systems in response to biological signals, such as exposure to human serum. In the article by Young et al. (contribution 2), the authors demonstrate that treatment of phenotypically susceptible strains in vivo can result in failure through this mechanism. This research identified a regulatory gene encoding a putative YhaK protein as essential to this process. The mutation of *yhaK* impaired efflux activity and increased intracellular drug accumulation, confirming its regulatory function. Notably, *yhaK* expression was significantly upregulated in human serum compared to standard media, highlighting its role in environmental adaptation and specific resistance to fluoroquinolones and tetracyclines in the human host.

The adaptability of *A. baumannii* extends further to traits that support biofilm formation, a key factor in its persistence on hospital surfaces and devices. Abd El-Rahman et al. (contribution 3) evaluated the contribution of efflux pump genes, particularly those belonging to the resistance-nodulation-division (RND) family, in biofilm development. While this study did not demonstrate a direct influence of these genes on biofilm formation, it pointed to the potential of further exploring efflux systems as therapeutic targets. Further understanding the role of efflux pumps in the formation of biofilms may offer future benefits in combating antimicrobial resistance and reducing environmental persistence.

In addition to exposure to antibiotics, clinical isolates of *A. baumannii* may also be exposed to various disinfectants and have developed resistance to many of these as well. Kelemen et al. (contribution 4) investigated the molecular basis of disinfectant resistance during an outbreak of CRAB in an intensive care unit in Hungary. This study demonstrated that modification of the disinfection protocol, specifically by replacing a quaternary ammonium compound (QAC)-based disinfectant (alkyl dimethyl benzyl ammonium chloride (C12-16 ADBAC), also known as MBF) with an alcohol-based disinfectant (10–20% 2-phenoxy-ethanol and 5–10% benzalkonium chloride, also known as IP), played a pivotal role in controlling the outbreak. In this study, six CRAB isolates from six individual patients all exhibited similar resistance profiles, showing resistance to carbapenems, aminoglycosides, and fluoroquinolones, while remaining susceptible to colistin. Genomic analysis of these isolates also revealed the presence of *qacE* and *qacEΔ1*, which encode transmembrane proteins responsible for QAC disinfectant tolerance. The products of these genes also contribute to broader biocide resistance by promoting efflux pump activity. The switch to an alcohol-based disinfectant, combined with stricter containment protocols, successfully ended the outbreak with no new infections reported, contrasting with more prolonged outbreaks when previous QAC-based protocols were utilized. This study underscores the importance of recognizing genetic mechanisms of disinfectant tolerance and suggests incorporating disinfectant susceptibility testing into revised infection control and antimicrobial stewardship programs.

Despite its historical use as a last-resort therapy due to nephrotoxicity, colistin has regained relevance for treating *A. baumannii* infections due to this pathogen’s resistance to nearly all other antibiotic classes, including carbapenems. The limited penetration of intravenous (IV) colistin into pulmonary tissues has, however, prompted exploration of alternative delivery methods. The study by Andrianopoulos et al. (contribution 5) investigated the efficacy of IV colistin in combination with high-dose nebulized colistin in treating ventilator-associated pneumonia secondary to *A. baumannii* bacteremia. Patients receiving this combination therapy showed significantly lower 7-day and 28-day mortality rates, greater sepsis resolution, and earlier vasopressor discontinuation compared to those treated with IV colistin alone. These findings reinforce the potential benefits of combination therapies as well as more targeted and localized antibiotic delivery methods to overcome tissue penetration barriers and combat MDR infections.

Lim et al. (contribution 6) investigated the potential use of fosfomycin in combination with sulbactam or colistin against clinical isolates of *A. baumannii* resistant to both antibiotics individually. While fosfomycin alone lacks reliable bactericidal activity against *A. baumannii*, its ability to inhibit early stages of cell wall synthesis makes it a valuable adjunct in combination regimens. This study demonstrates significant in vitro synergy when fosfomycin was combined with sulbactam, particularly in isolates that had previously shown resistance to sulbactam monotherapy. This synergistic effect is believed to arise from fosfomycin’s disruption of peptidoglycan biosynthesis, which may enhance sulbactam’s ability to bind to penicillin-binding proteins. Additionally, this combination therapy was noted to reduce the minimum inhibitory concentrations of both drugs, potentially restoring clinical efficacy. These findings are critical in the context of limited treatment options for drug-resistant *A. baumannii*, underscoring the importance of re-evaluating older antibiotics like fosfomycin within strategic combination therapies to overcome complex resistance phenotypes and improve patient outcomes.

A detailed review of current therapeutic approaches for CRAB infections, particularly in light of emerging *β*-lactam agents, is provided by Rafailidis et al. (contribution 7). This review emphasizes the limitations of traditional antibiotics such as colistin and tigecycline when used as monotherapies. Colistin, though frequently deployed as a last-resort therapeutic, poses significant challenges due to its nephrotoxicity and limited penetration into pulmonary tissues when delivered intravenously. Tigecycline, while offering utility in the treatment of soft tissue infections, is less effective in the treatment of bacteremia because it does not achieve high serum concentrations. The authors argue for the increased efficacy of combination regimens, especially those incorporating agents with complementary mechanisms of action. Notably, they highlight the potential benefits of treating with sulbactam–durlobactam, a novel *β*-lactam/*β*-lactamase inhibitor pairing, which demonstrates intrinsic activity against *A. baumannii* and overcomes class D *β*-lactamase-mediated resistance. Another promising agent discussed is cefiderocol, which exhibits high stability against all four classes of *β*-lactamases and uses a siderophore-mediated entry system to reach its target.

Building on the aforementioned considerations, the review by Serapide et al. (contribution 8) examines the pharmacokinetic and pharmacodynamic (PK/PD) challenges associated with treating CRAB, particularly in critically ill patients. This review demonstrates how suboptimal tissue concentrations of antibiotics, even when used to treat phenotypically susceptible isolates, can lead to therapeutic failure. This review reinforces the necessity of optimizing dosing strategies, especially for agents like cefepime and tigecycline, to ensure effective drug levels at infection sites. This review also underscores the clinical potential of cefiderocol, citing its effective penetration and unique mechanism of action wherein bacterial iron uptake pathways are exploited to gain access to the periplasmic space. In addition to its broad stability against *β*-lactamases, cefiderocol is associated with lower mortality rates when compared to colistin-based therapies. This review reemphasizes concerns over emerging resistance and the importance of antibiotic stewardship to preserve the efficacy of limited therapeutic interventions.

## 3. Discussion and Conclusions

This Special Issue underscores the complexity and clinical urgency of combating *A. baumannii*, particularly CRAB strains. The featured contributions highlight a multifaceted public health challenge, where this bacterium’s ability to persist in healthcare settings, resist both antibiotics and disinfectants, and rapidly adapt to environmental pressures, allows this pathogen to continually overcome current countermeasures [1,5]. Collectively, these studies reinforce the importance of genomic surveillance in identifying resistance mechanisms and tracking the spread of high-risk *A. baumannii* lineages to inform region-specific treatment protocols [7]. Insights into adaptive resistance, such as serum-induced efflux mechanisms and increased environmental tolerance, highlight the fact that reduced antibiotic clinical efficacy is driven not only by classical antibiotic resistance mechanisms, but also by dynamic gene regulation and stress response pathways. The ability of *A. baumannii* to adapt to various environments is strengthened by the accumulation of resistance determinants through both horizontal and vertical gene transfer, enabling rapid dissemination of resistance within and across bacterial populations. Genomic characterization of *A. baumannii* is therefore essential to decipher the genetic makeup of resistant strains and select more effective, isolate-specific treatment strategies [8]. Ultimately, infection control strategies that consider this pathogen’s resistance to both antibiotics and disinfectants, whose resistance mechanisms could be shared, are critically needed.

While disinfection protocols are essential for curbing the spread of *A. baumannii*, mitigation of this pathogen’s ability to establish an infection and the achievement of improved clinical outcomes demand a more comprehensive understanding of the organism’s pathogenicity and resistance mechanisms. The success of *A. baumannii* as a nosocomial pathogen lies in its multifaceted virulence strategies, which include the production of *β*-lactamases, alterations in outer membrane proteins, activation of multidrug efflux pumps, and robust biofilm formation. These mechanisms work synergistically to enhance the organism’s survival in hostile environments, contributing to its persistence and complicating eradication efforts [9]. These challenges underscore the necessity for continuous evaluation of current therapeutic strategies, deeper investigation into the potential of combination therapies, and the development of novel antimicrobial agents.

In response to difficult-to-treat resistant (DTR) and PDR *A. baumannii*, combination therapy has emerged as a cornerstone strategy. Empirical regimens often begin with high doses of ampicillin–sulbactam or tigecycline. Polymyxins such as colistin are also commonly employed, despite their known limitations, including nephrotoxicity and poor pulmonary penetration. Newer agents like sulbactam–durlobactam (SUL/DUR) and cefiderocol have shown considerable promise in treating CRAB infections. SUL/DUR has demonstrated favorable activity and may be a preferable option for critically ill patients, particularly when other alternatives are limited [10]. In critically ill patients, optimizing PK/PD parameters is vital; for example, transitioning from intermittent to continuous infusion of *β*-lactams has been associated with improved clinical outcomes [11]. These strategies highlight the importance of individualized therapy guided by antimicrobial susceptibility profiles and patient-specific factors, reinforcing the critical role of antimicrobial stewardship programs.

In summary, this Special Issue offers a comprehensive overview of the attributes and challenges presented by A. baumannii. These studies advance our understanding of available treatments and shed light on mechanisms that drive immune evasion and lead to antibiotic failure. Emphases on early detection and surveillance strategies for both antibiotic and disinfectant resistance highlight emerging approaches in infection control. Ultimately, the contributions within this Special Issue aim to drive continued research into novel therapeutic development, optimization of current drug combinations, and identification of genetic determinants that drive resistance and persistence. Only through interdisciplinary collaboration between fields such as basic microbiology, pharmacology, and epidemiology can we outpace the evolving threat posed by this highly adaptive pathogen.

## References

[1] CDC (2019). Antibiotic Resistance Threats in the United States, 2019.

[2] Boucher H.W., Talbot G.H., Bradley J.S., Edwards J.E., Gilbert D., Rice L.B., Scheld M., Spellberg B., Bartlett J. (2009). Bad bugs, no drugs: No ESKAPE! An update from the Infectious Diseases Society of America. Clin. Infect. Dis..

[3] Howard A., O’Donoghue M., Feeney A., Sleator R.D. (2012). *Acinetobacter baumannii*: An emerging opportunistic pathogen. Virulence.

[4] Nowak J., Paluchowska P. (2016). *Acinetobacter baumannii*: Pathogenesis, antimicrobial resistance, and treatment options. Adv. Clin. Exp. Med..

[5] WHO (2017). Global Priority List of Antibiotic-Resistant Bacteria to Guide Research, Discovery, and Development of New Antibiotics.

[6] Vrancianu C.O., Gheorghe I., Czobor I.B., Chifiriuc M.C. (2020). Antibiotic resistance profiles, molecular mechanisms and innovative treatment strategies of *Acinetobacter baumannii*. Microorganisms.

[7] Higgins P.G., Dammhayn C., Hackel M., Seifert H. (2010). Global spread of carbapenem-resistant *Acinetobacter baumannii*. J. Antimicrob. Chemother..

[8] Wiradiputra M.R.D., Thirapanmethee K., Khuntayaporn P., Wanapaisan P., Chomnawang M.T. (2023). Comparative genotypic characterization related to antibiotic resistance phenotypes of clinical carbapenem-resistant *Acinetobacter baumannii* MTC1106 (ST2) and MTC0619 (ST25). BMC Genom..

[9] Peleg A.Y., Seifert H., Paterson D.L. (2008). *Acinetobacter baumannii*: Emergence of a successful pathogen. Clin. Microbiol. Rev..

[10] Zhang S., Di L., Qi Y., Qian X., Wang S. (2024). Treatment of infections caused by carbapenem-resistant *Acinetobacter baumannii*. Front. Cell. Infect. Microbiol..

[11] Roberts J.A., Abdul-Aziz M.-H., Davis J.S., Dulhunty J.M., Cotta M.O., Myburgh J., Bellomo R., Lipman J. (2016). Continuous versus intermittent β-lactam infusion in severe sepsis. A meta-analysis of individual patient data from randomized trials. Am. J. Respir. Crit. Care Med..

